# Clival Defect Resulting in Spontaneous Cerebrospinal Fluid Rhinorrhea: Case Report and Review of Literature

**DOI:** 10.1155/2023/3205191

**Published:** 2023-07-24

**Authors:** Maryam Aljawi, Mahdi Shkoukani

**Affiliations:** Otolaryngology Head and Neck Surgery Cleveland Clinic, AbuDhabi, UAE

## Abstract

Spontaneous cerebrospinal fluid (CSF) rhinorrhea develops in patients without any history of trauma. Multiple factors have been theoretically debated. Also, localizing the defect may result in a challenge for the rhinologist. The common locations are the cribriform plate and the lateral recess of the sphenoid. Clival CSF rhinorrhea is rare, and only few cases have been reported so far. A 52-year-old female presented to the otolaryngology clinic with 7 years of history of left-side clear fluid rhinorrhea as a drop, which progressed to be runnier after she had pneumonia with severe cough secondary to COVID-19 infection. CSF was confirmed by a beta-2-transferrin test. During the perioperative evaluation, she developed meningitis which was treated with IV ceftriaxone and IV vancomycin antibiotics. The magnetic resonance imaging (MRI) and computerized tomography (CT) scan showed clival defect with pseudomeningocele which was initially not easy to see on CT. The patient underwent an endoscopic approach to the skull base to repair the defect with a pedicled septal flap. Also, a lumbar drain with intrathecal fluorescein administration was utilized. The postoperative course was uneventful without any complications. There was no evidence of recurrence with a 9-month follow-up postoperatively.

## 1. Introduction

Etiologies for CSF rhinorrhea are divided into traumatic and nontraumatic causes; nontraumatic causes are then subdivided based on the intracranial pressure (ICP), either normal or raised [1]. Spontaneous CSF rhinorrhea is categorized under normal ICP or elevated ICP [1]. Spontaneous CSF rhinorrhea accounts for 40% of all CSF rhinorrhea, and it is considered a rare presentation; the most identified defect sites are the cribriform plate and the lateral wall of the sphenoid [2]. Clival defect is very rare, and based on a literature review, only 24 cases have been reported so far since 1980. Patients with CSF rhinorrhea usually present with one-sided clear fluid rhinorrhea that worsens with bending forward, and the most used and accurate investigation to identify that the fluid is CSF is beta-2-transferrin which has a specificity of 98% and a sensitivity of 94% [3]. CT scan and MRI are used to help in localizing the site of the defect, the presence of meningocele or meningoencephalocele, and the presence of increased intracranial pressure by having an empty sella or arachnoid pits [1]. In cases of increased intracranial pressure, a lumbar drain can be used and sometimes, it is used intraoperatively with intrathecal fluorescein injection to help identify the site of the defect during the endoscopic approach if other images are not helpful in localizing the defect [1]. Multiple techniques have been described in repairing skull-based defects. There are different grafts that can be used to close the defect, namely, synthetic, xenograft, or autograft. Also, studies have shown that vascularized flaps are superior in the setting of large dural defects or high-flow CSF leaks [4, 5]. CSF rhinorrhea should be repaired and managed to prevent serious complications such as meningitis [1].

## 2. Case Report

**Figure 1:**
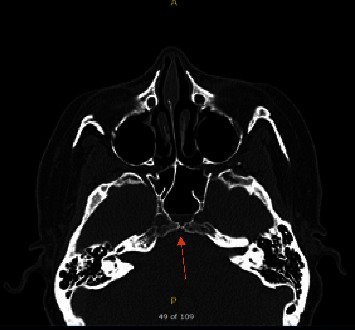
CT sinus without contrast axial cut showing the air-fluid level in the sphenoid sinus and defect in the clivus (see arrow).

**Figure 2:**
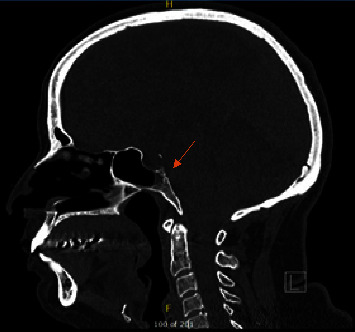
CT sinus without contrast sagittal cut showing the air-fluid level in the sphenoid sinus and defect in the clivus (see arrow).

**Figure 3:**
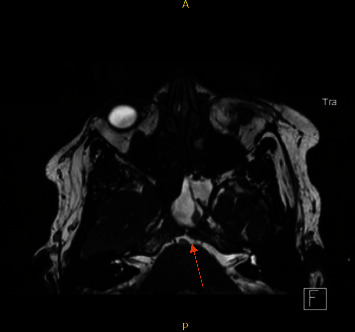
MRI skull base with IV contrast axial cut showing fluid accumulation in sphenoid communicating with clival defect (see arrow).

**Figure 4:**
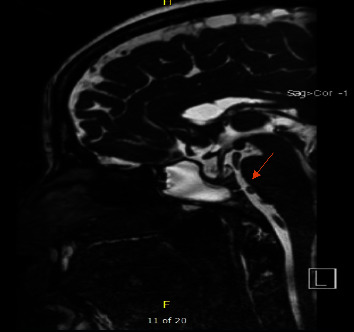
MRI skull base with IV contrast sagittal cut showing fluid accumulation in sphenoid communicating with clival defect (see arrow).

**Figure 5:**
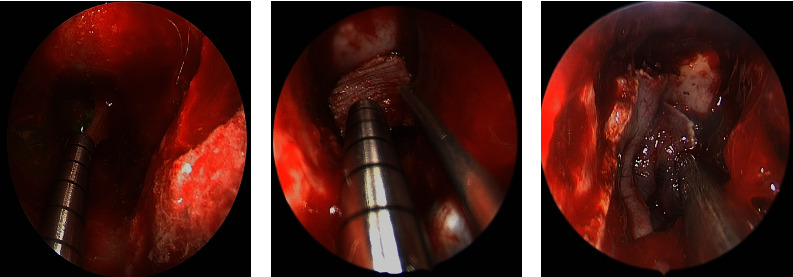
(a)–(c): (a) Fluorescein dye coming from the defect site. (b) Bovine pericardium graft placed over the defect followed by fibrin sealant. (c) Pedicled septal flap placed over the defect.

**Figure 6:**
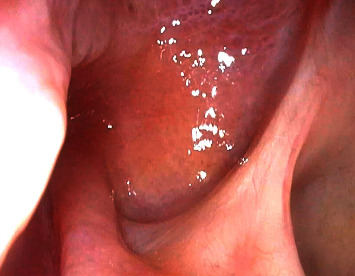
10 months postoperative endoscopic picture showing healed skull base.

A 52-year-old female, known to have hypertension and obesity with BMI 33, presented to the otolaryngology clinic complaining of 7-year history of left-sided clear rhinorrhea started as a drop with on and off headaches. Then, 3 months before presentation, she had developed pneumonia secondary to COVID-19, which resulted in severe cough, and at that time, she noticed her rhinorrhea became worse, which aggravated with bending forward. Her clear fluid was tested by beta-2-transferrin which confirmed CSF. Her CT skull base without contrast (Figures 1 and 2) and MRI skull base with IV contrast (Figures 3 and 4) showed clival defect with pseudomeningocele and no signs of increased ICP. The CT scan was performed first, and it was a challenge for the radiologist to read it and that is when an MRI was recommended. During her perioperative study, she developed meningitis that was confirmed with lumber puncture, which was treated with IV ceftriaxone and vancomycin without complications or neurological deficit for 14 days. After her meningitis recovery, she underwent an endoscopic approach to the skull base to repair the defect. Also, a lumbar drain with intrathecal fluorescein administration was utilized. There was normal opening pressure. Intraoperatively, fluorescein confirmed the leak coming from the clivus and there was no other leak identified (Figure 5(a)). The defect was repaired with multilayer closure. An onlay bovine pericardium graft was placed followed by a fibrin sealant (Figure 5(b)). Then, a pedicled septal flap was placed over it (Figure 5(c)). Postoperatively, the lumbar drain was kept in place for 6 days at 10 ml/hr. On her 9-month follow-up visit, the patient was doing well with no evidence of recurrence (Figure 6).

## 3. Discussion

There are multiple factors that could possibly lead to spontaneous CSF leak, anatomical and physiological [1]. Anatomical leak has been related to the bone and meninges, and physiological leak is related to the pressure gradient [1]. In relation to the most common areas of spontaneous CSF rhinorrhea, we can see that it involves the cribriform plate, which is a thin bone with variability in thickness between individuals in studies related to the human skull [6]. The clivus is considered rare because it is a thick bone not affected by physiological changes [7]. It is believed that 3 theories could explain it to happen. One might be related to ecchordosis physaliphora found in 0.5–2% of cases. It is a benign notochordal remanent found in the intradural space in either the clivus or the sacrum, it does not cause bone invasion but can form CSF fistula, and due to physiological changes related to intracranial pressure and basilar artery pulsation behind the defect can lead to dural erosion and the leak to happen [8]. Second, it could be related to sphenoid pneumatization [9]. There are 3 types of sphenoid pneumatization, namely, conchal, presellar, and sellar. Excessive pneumatization for the sellar type could lead to the thinning of the clival bone and make it prone to physiological changes leading to CSF leak [9]. Third, it is believed that during embryological developments, the skull bones are fused at different points by synchondrosis which is a hyaline cartilaginous joint [9]. The fusion between spheno-occipital is the last to be fully fused ranging between 14 and 25 years of age; so, incomplete fusion with the contribution of physiological factors can lead to CSF leak [9].

Given its rare location, it is considered a challenge in terms of diagnosis and possibly surgical management. There were cases reported as being missed, leading the patient to present with recurrence because they were operated on without knowing the exact location of the defect and assuming the possibility of it being in the common areas [10]. The standard images that are used to identify the site of defect are CT and MRI, which help identify the bony and soft tissue changes, respectively [1]. Intraoperatively, it might be difficult to identify the site of the defect, especially in cases with low-flow CSF leaks [1]. Lumbar drain with intrathecal fluorescein injection is not widely used, but in the rare clival defect, it might help better localize the site and potentially rule out other sites of leak [1]. Surgical challenges in repairing clival defects arise from choosing the appropriate graft vs. flap which may depend on surgeon preference and the flow rate of the leak [4, 5]. Most studies showed that reconstructing these defects mainly depended on surgeon preference and the availability of allograft/xenograft in the facility [11]. It is important to keep the cases of increased intracranial pressure in consideration. Having a normal pressure at the time of lumbar puncture does not necessarily rule out intracranial hypertension as the patient has lost a significant amount of fluid. These cases need to be monitored postoperatively very closely and may need to be treated with acetazolamide. Managing CSF leak is crucial to prevent complications such as meningitis and brain abscess that could be fatal.

## Data Availability

The data that support the findings of the study are available from the corresponding author upon request.
